# Executable biochemical space for specification and analysis of biochemical systems

**DOI:** 10.1371/journal.pone.0238838

**Published:** 2020-09-11

**Authors:** Matej Troják, David Šafránek, Lukrécia Mertová, Luboš Brim

**Affiliations:** Systems Biology Laboratory, Masaryk University, Brno, Czech Republic; Gulf University for Science & Technology (GUST), and University of Jena, GERMANY

## Abstract

Computational systems biology provides multiple formalisms for modelling of biochemical processes among which the rule-based approach is one of the most suitable. Its main advantage is a compact and precise mechanistic description of complex processes. However, state-of-the-art rule-based languages still suffer several shortcomings that limit their use in practice. In particular, the elementary (low-level) syntax and semantics of rule-based languages complicate model construction and maintenance for users outside computer science. On the other hand, mathematical models based on differential equations (ODEs) still make the most typical used modelling framework. In consequence, robust re-interpretation and integration of models are difficult, thus making the systems biology paradigm technically challenging. Though several high-level languages have been developed at the top of rule-based principles, none of them provides a satisfactory and complete solution for semi-automated description and annotation of heterogeneous biophysical processes integrated at the cellular level. We present the second generation of a rule-based language called Biochemical Space Language (BCSL) that combines the advantages of different approaches and thus makes an effort to overcome several problems of existing solutions. BCSL relies on the formal basis of the rule-based methodology while preserving user-friendly syntax of plain chemical equations. BCSL combines the following aspects: the level of abstraction that hides structural and quantitative details but yet gives a precise mechanistic view of systems dynamics; executable semantics allowing formal analysis and consistency checking at the level of the language; universality allowing the integration of different biochemical mechanisms; scalability and compactness of the specification; hierarchical specification and composability of chemical entities; and support for genome-scale annotation.

## Introduction

Modelling complex systems in systems biology has to be conducted at several levels of abstraction that reflect well the known information [1]. At every level, the system has to be described rigorously in a formal language that allows avoiding misunderstanding and ambiguous interpretations. The more complex the system is, the harder it is to describe it rigorously and compactly in a way understandable for users outside of computer science. Properties required to be attained by such language come from the needs of effective execution of the general workflow typically employed in systems biology research which relies on a combination of model- and data-driven procedures. In particular, the target properties of the workflow mirror the general needs of accessibility, interoperability, and reusability, as discussed in [2]. To address the aim of bringing these properties into a single platform supporting most of the typical systems biology procedures, we have introduced the *Comprehensive Modelling Platform* (CMP) [3]—an online platform for modelling of biological processes that combines, in a unique way, experimental data with mathematical modelling, state-of-the-art data annotation standards, and graphical presentation enabling accessibility to users with different expertise. Development of such a platform shows an urgent need for having a sufficiently abstract formal language for unambiguous mechanistic description of biological processes that will glue together known information, existing mathematical models, and new experimental data while supporting model integration and prediction of new hypotheses based on integrated models.

In general, an ideal biochemical system specification language that can be sufficiently employed in systems biology practice has to be *hierarchical*, *executable*, and it has to inherently support widely accepted standards for biological *data annotation*. Hierarchical description allows expressing individual system components at different levels of detail. Since not all biochemical structures are known in detail, the language has to support *expression of partial knowledge*. On the other end, executability is based on associating the model description with appropriate formal (mathematical or programming) structures representing the dynamics of the model. These structures enable simulation and global (exhaustive) analysis of the dynamics or they help to reveal inconsistencies in the model. Support of annotation standards is necessary to enable reusability of models, e.g., allowing integration with other models—a critical step on the challenging journey towards comprehensive models of tissues or entire organisms.

Traditional approaches used to describe biochemical systems are: (i) a *chemistry approach* employing “mechanical” descriptions by chemical reactions or (ii) a *mathematical approach* using ordinary differential equations or other mathematical formalisms. An advantage of chemical reactions over mathematical equations is the fact that chemical reactions are composable to some extent, easy to understand, and widely used in chemistry and biology. Moreover, they can be directly assigned an executable semantics that enables to globally explore dynamics of systems of reactions. Additionally, there are methods supporting the automated generation of mathematical models from chemical reactions. In general, chemical reactions can be understood as a universal (semi-)formal language of chemistry [4].

**Fig 1:**
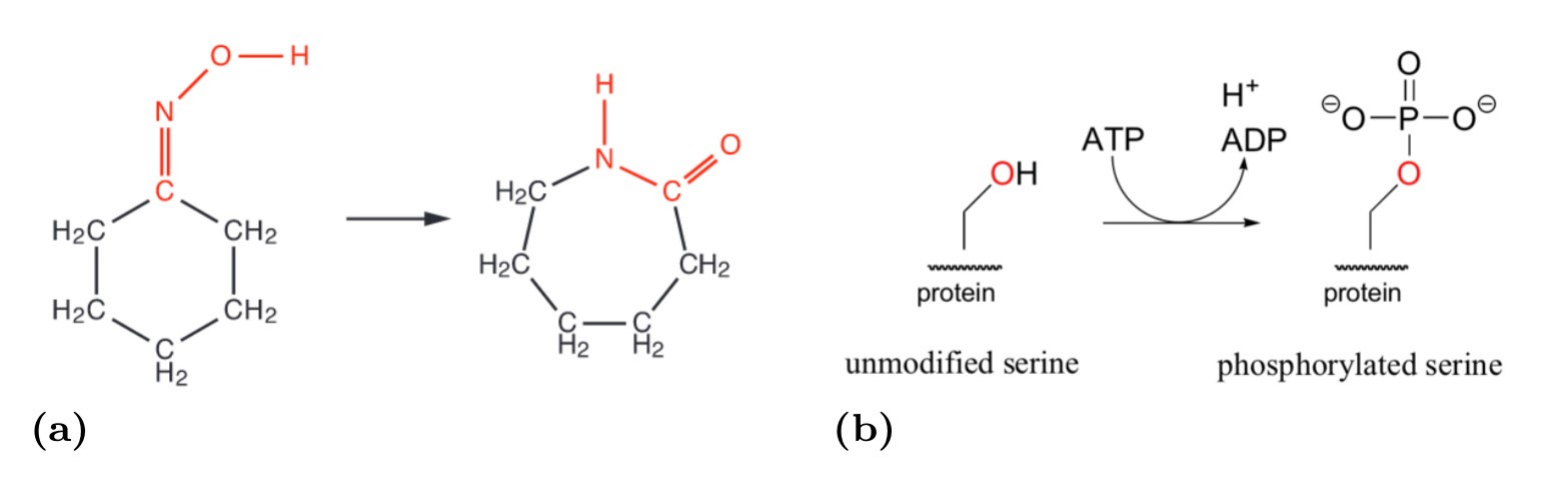
Examples of chemical reactions. (A) An example of *elemental* chemical reaction. The description of interacting species is given on molecular level, providing exact specification of *how* the species is transformed. (B) An example of *general* chemical reaction. A protein is being phosphorylated on a serine residue. The particular interaction is described in detail (which does not generally hold for this type of reactions) but the molecular details of the entire species is abstracted, since the focus is put only on the interaction itself.

There are two basic levels of abstraction that can be achieved with chemical reactions: the low (detailed) level provided by *elemental* chemical reactions showing how particularly are chemical species transformed on a molecular scale, and *general* (or *stepwise*) chemical reactions, that describe higher order transformations representing multiple elemental processes [5] (for an example, see Fig 1). The reason for the usage of general chemical reactions is the fact that for the transformation of more complicated chemical objects (e.g. proteins) the detailed description is not feasible and a suitable abstraction needs to be applied such that the process can be described rigorously yet not on the detailed molecular scale.

A significant problem of both chemistry and mathematical approaches is the *scalability*, understood at two different levels: scalability of the model description (avoiding combinatorial explosion at the syntactic level, i.e., the necessity of repeating equivalent mechanisms for an exponential number of entities such as molecules independently phosphorylated at several sites) and scalability of the model execution (avoiding combinatorial explosion at the semantics level, i.e., the necessity to generate all functional states of the model dynamics). Even when the formulation of a model does not run into syntactic scalability problems, the execution or simulation might be infeasible [6], thus critically affecting the potential of automated analysis. The so-called *computational approach* [7, 8] offers a scalable model description by abstracting the information about individual model components and interactions. The scalability at the semantics level still remains to be a challenge. In this paper, we primarily focus on the model description level while leaving improvements of the computational aspects for future work.

A promising computational approach addressing some of the above mentioned issues is provided by *rule-based modelling* [9, 10] and process-algebraic frameworks [7, 11, 12]. Rule-based models make a natural extension of the mechanical reaction-based models used in chemistry by introducing abstraction of details forming the behavioural *patterns*. Instead of operating with objects, rule-based frameworks operate with *types* that allow avoiding the combinatorial explosion that occurs when underlying objects are specified directly. The semantics of the model is given in terms of *rules* defined on given types. Process-algebraic approaches make a theoretical basis for rule-based modelling. Object types are considered as dynamical processes interacting via communication channels. An important advantage of rule-based and process-algebraic approaches is that mathematical models can be automatically generated from them. In particular, instead of relying on a single mathematical formalism, different mathematical models can thus be obtained for a given model (e.g., ODEs [13], PDEs [14], chemical master equation or continuous-time Markov chains [15, 16], reaction-diffusion systems [17], etc.).

The main examples of rule-based languages are: BNGL [10] and Kappa [9] supporting the detailed expression of molecule binding; Chromar [18] associating objects with physical attributes allowing to describe observed system quantities; rxncon [6, 19] focusing on regulatory interactions and allowing construction of rules from experimental evidence; LBS [20] and LBS-*κ* [21] enriching rule-based framework with modularity; PySB [22] embedding BNGL into Python; BioCHAM [23] explicitly separating rules from their mathematical semantics. An important work is ML-rules [24], which allows linkage of species with unique values in a highly flexible manner (e.g. hyperedges) and introduces the concept of nested species. In the field of the process algebraic approach, the most representative languages are: BioSPI [12] and SPiM [25] using asymmetric binary synchronisation primitives to capture interactions; BioPEPA [11] embedding chemical reactions directly into the process-algebraic framework.

It is also worth noting that the rule-based approach has been recently adopted by SBML in terms of the package multi [26]. It employs XML in order to represent the hierarchical structure of objects and modularity of rules. It moves the rule-based paradigm towards a standard format for describing biological systems. However, it does not directly solve the executability and advanced analysis issues that make an important aspect of rule-based frameworks. At the level of annotation, the solution for SBML multi is provided in [27].

**Fig 2:**
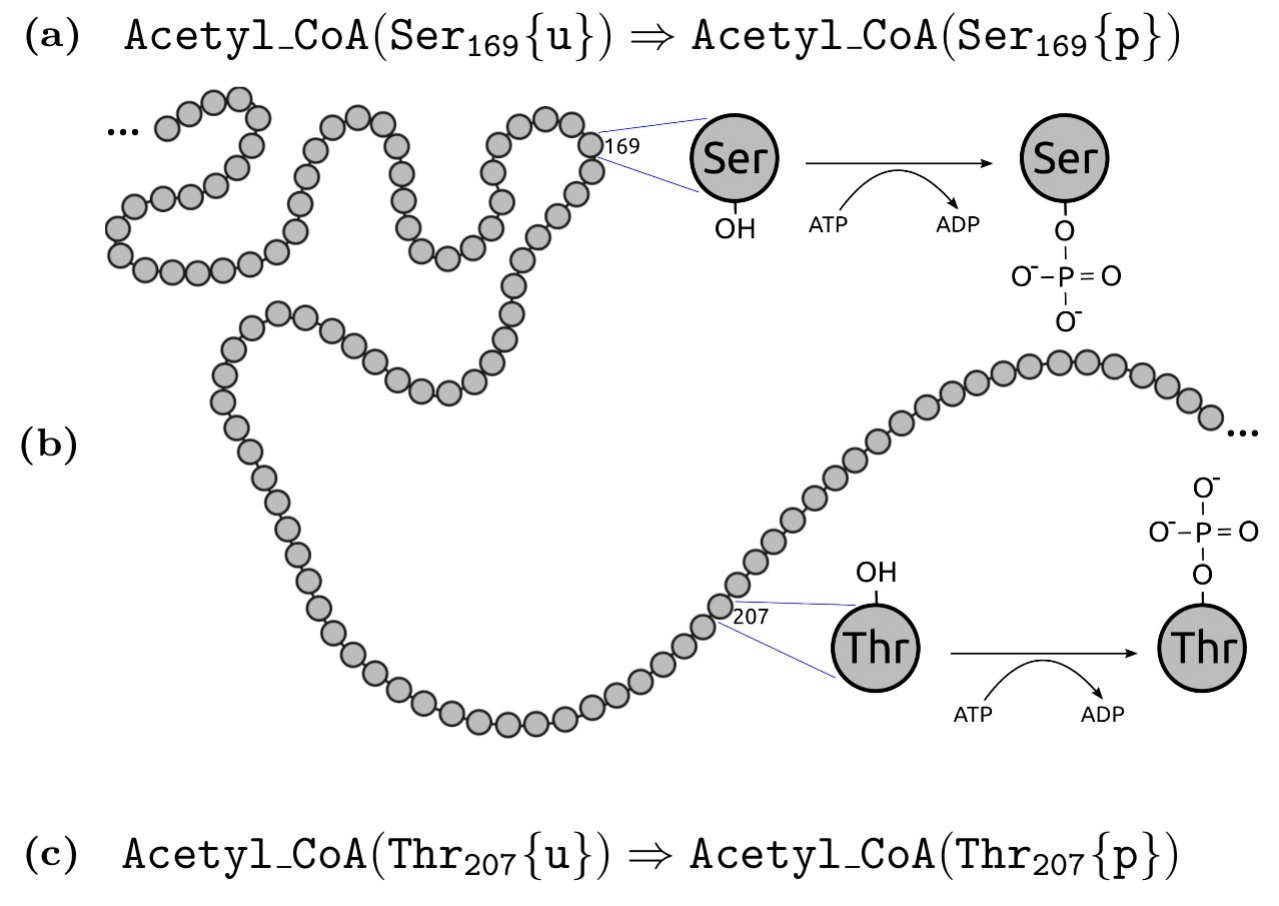
(B) A schematic description of a part of primary of Acetyl-CoA carboxylase (UniProt∷O00763). Two amino acid residues of modelling interest are highlighed—a serine on position 169 and a threonine on position 207—where both of them can be modified by an independent phosphorylation process. (A) (resp. (C)) The rule-based abstraction allows to write a *rule* which focuses purely on the particular phosphorylation of serine (resp. threonine) residue, omitting all other functional parts of the protein which do not play any role in this post-translational modification.

An unavoidable problem of rule-based modelling is the tradeoff between the compactness and the level of detail expressed. A good example is Kappa [28] that employs a very detailed description of interactions provided by *bonds* between species. This allows specification of a large scale of entities ranging from small molecules to complex proteins provided that interactions among them are modelled in such detail. In general, in rule-based modelling, it is needed to fine-tune the required level of abstraction for the concrete modelling aims. This affects important design decisions for a particular language. As shown in Fig 2, in some modelling scenarios, it appears to be useful to abstract from details of molecules binding.

Despite the fact that rule-based languages provide a very flexible solution based in chemistry approach while allowing to automatically transfer to the mathematical approach, our experience from several systems biology projects EC-MOAN, e-photosynthesis, e-cyanobacterium, ISBE is that the direct use of the mathematical approach, i.e., ODEs, is still a typical scenario. Lack of precise annotation of some previously created ODE models requires a non-trivial effort before these models can be reused, extended, or integrated. Moreover, even well-annotated ODE models coming from curated sources (e.g., [29, 30]), cannot be easily re-interpreted due to the presence of relatively complicated equations of higher-order kinetic laws. Integration of such models is difficult due to possible incompatibilities among different levels of detail employed. Moreover, complicated mathematical models containing large sets of parametrised equations are error-prone and it is a non-trivial problem to check their correctness and internal consistency. Standards like SBML [31] or CellML [32] bring a significant help. However, the mentioned problems cannot be completely eliminated due to the inherent characteristics of ODE models.

Existing rule-based formalisms offer specific technical solutions to modelling and description of biological processes ranging from compact representation to various analyses methods. However, none of them, in the current form, satisfies all the requirements needed for reproducible and reusable comprehensive modelling that would be directly accessible to users from life sciences. In particular, the problem is to provide enough flexibility and an adequate level of abstraction allowing to satisfactorily integrate the existing mathematical models, experimental data, and databases of biological knowledge by projecting them onto the sufficiently abstract and user-accessible rule-based description that is mechanistic, rigorous and glues all pieces of information together. To tackle this problem, we have introduced the idea of rule-based *Biochemical Space* (BCS) [3] that semi-formally and in a unified way annotates the mechanistic information encoded (and often “encrypted”) in mathematical models while making a bridge between the mathematical models and the biological knowledge. Such an approach has three main advantages. First, fully manual direct annotation of mathematical models can be replaced with semi-automated mapping to well-annotated BCS. Second, inconsistencies and overlaps between several mathematical models can be automatically discovered by mapping them to BCS. Third, BCS can be understood itself as a comprehensive computational model that can be in future used for analysis or automated generation of mathematical models. The latter issue gives the main motivation for making BCS a fully formal framework supporting executability and automated analysis. These points give the requirements we have attempted to address by introducing the *Biochemical Space Language* (BCSL) that combines the advantages of rule-based modelling with the simplicity of chemical reactions [33].

In this paper, we present BCSL as an advanced formal method that extends the theoretical results achieved in our previous research and unifies them into a a single comprehensive framework in a unique way. Moreover, we embed BCSL into the general context of the Comprehensive Modelling Platform. On the technical side, the definition of the language semantics is completely reformulated in terms of set theory in contrast to the infeasible process algebra approach originally employed in [33]. From the syntactic perspective, the key updates are the introduction of nesting operator, variables, and complex aliases. In addition, we provide a detailed comparison of BCSL with the main representatives of state-of-the-art rule-based languages. In general, the complete framework of BCSL presented here displays a robust solution to formal specification of biological processes that adopts the following aspects in a single solution: *user-accessibility* (easy to share and maintain); *executability* (formal executable semantics is defined allowing some static analysis and automated consistency checking of the specification); *universality* (principally different cellular processes can be sufficiently combined in a single specification); *scalability* (combinatorial explosion of the description is avoided); *hierarchy* (object types are described hierarchically allowing compositional assembly from simpler structures); *annotation* (uses SBML-compliant genome-scale annotation standards allowing connecting the object types and rules with bioinformatics knowledgebases). On the practical side, we demonstrate the usability of BCSL on the specification of several biological processes. First, we show how various processes of cyanobacteria can be expressed and how BCS annotation can help in the understanding of mathematical models. Second, we explore the universality of BCSL on an example of mammalian cells signalling pathway. More specifically, we show how processes on a different level of abstraction can be expressed together in a single BCSL specification. Finally, we show how several BCSL-based static analysis techniques can help to reveal problems in the biological processes modelling workflow.

The primary prototype of BCSL has been introduced in [33] with the formal semantics defined at the top of Kappa. However, BCSL aims at higher-level abstraction than Kappa that focuses on morphisms between protein binding sites. Therefore the Kappa-based formulation of BCSL does not fit well the aims of the general modelling framework. In [34], we have defined hierarchical and composable object types and rules as standalone structures and we avoided any loss of information caused by transforming them into another language. That way, we have obtained qualitative executable semantics of rules directly at the level of the language thus making a base for unique qualitative analysis tasks specific for the considered level of abstraction. In this paper the semantics is further reformulated in terms of set theory making the semantics more comprehensible and bringing several new operators into the formal language – nesting, variables, and complex aliases. All ideas employed during the language development are finally formalised in the final version of BCSL presented in this paper. BCSL is currently implemented in the first instance of the Comprehensive Modelling Platform, e-cyanobacterium.org, focused on cyanobacteria modelling [35]. The platform provides software for maintenance of BCSL specifications. Besides using BCSL for qualitative modelling as presented in this paper, we aim to shift BCSL towards a technology that will support also quantitative models. First steps in that direction are currently achieved by introducing qBCSL [36] (the quantitative extension of BCSL) and developing the software tool eBCSgen [37] for specification and analysis of qBCSL models.

The closest languages reflecting the required level of abstraction are BioCham [23] and LBS [20]. Both languages focus on defining objects at a single level of detail and they do not provide sufficient flexibility, annotation mechanisms and hierarchical description required for the purpose of the general modelling platform as mentioned above. Moreover, the BioCham language is tightly connected with the BioCham tool. Apparently, various aspects of different languages are needed to be combined and further extended in order to sufficiently describe heterogeneous biophysical processes as expected in the general framework. That is addressed with BCSL in this paper.

The paper is structured as follows: In the first section, we introduce the Comprehensive Modelling Platform with its respective parts; in the second section, the Biochemical Space Language and its semantics is described intuitively and several static analysis techniques are presented; in the next two sections, key advantages of the language are demonstrated on several case studies and a comparison with other rule-based formalisms is given.

## Comprehensive modelling platform

*Comprehensive Modelling Platform* (CMP), introduced in [3], is an online platform providing tools for public sharing, annotation, analysis, and visualisation of dynamical models and *wet-lab* experiments related to domain-specific systems. The platform is unique in integrating abstract mathematical models with a precise biochemical description provided in a rule-based formalism. The general aim is to stimulate collaboration between experimental and computational systems biologists to achieve better understanding of the domain-specific system. The general platform is currently under development Comprehensive Modelling Platform GitHub project. However, the first domain-specific instance of CMP, e-cyanobacterium.org, is already available online as a proof of concept e-cyanobacterium.org. It serves as an international modelling hub for systems biology of cyanobacteria.

### Biochemical space

The platform consists of several dedicated modules, all connected to a central module—*Biochemical Space* (BCS)—the backbone of the platform [34]. BCS provides formal description and annotation of the biological system and it is based on the hierarchy of selected biological processes. It is accompanied with schemas representing relevant biological processes in the context of the modelled system. For each process, there are present the relevant models, chemical agents, and rules. Presentation of every process includes detailed information and links to the relevant internal and external sources.

BCS provides a well-described biological background for mathematical models of processes taking place in a specific organism. Complete BCS provides a connection between existing ontologies and mathematical models. BCS has a *human-readable* format which can be easily edited in a dedicated editor and visualised within the platform. BCS is formed by two parts—a set of *agents* and a set of *rules*.

When building the BCS, the emphasis is put on well-defined and complete annotations. Links to the relevant ontologies must be specified for each agent and rule. Unique IDs provided by ontologies can help to automatically detect duplicities. IDs are also used to create hypertext links to related ontologies on the web, thus providing one part of the already mentioned connection between ontologies and models. Currently, links to KEGG [38], ChEBI [39], UniProt [40], CyanoBase [41], and other databases are supported. A single agent or a rule can have multiple links to several external databases. An example is the presence of a particular agent in UniProt as well as in CyanoBase.

### Model repository

Model repository is a collection of mathematical models describing particular parts of the biological processes. Each model is implemented as a set of ordinary differential equations generated from the model reaction network. The model is associated with some parameter value sets (*data sets*) that enable simulation in particular biologically-relevant scenarios.

Models are integrated within BCS. In particular, each model component should be related to some BCS agent and each model reaction should be related to some BCS rule.

The problem can occur when we try to map a model which uses more detailed description of the system than BCS does. However, we simply allow relating multiple model objects to BCS objects and vice versa. For example, multiple model equations might be mapped on a single BCS rule denoting the fact that the process represented by the rule can be described in more details by the equations. On the other hand, the model might neglect some details. For example, a reaction can be mapped on multiple rules with similar meaning than in the previous case.

The implemented model includes complete biological annotation of all components and equations that is provided by the mapping to BCS. This can help to find connections and overlaps among models. The model can be exported in SBML format, annotated by URIs preserving the mapping to BCS database using established resolving system Identifiers.org [42].

### Experiments repository

Experiments repository is a module for storage and presentation of time-series data from wet-lab experiments. Every experiment is well-grounded by precise description (device, medium, organism, etc.) and appropriate annotations. Experiments are structured—several time series data can be attached to a single experiment. Every time series targets a specific list of measured substances together with time stamps of the individual measurements. Time series can be visualised in a chart.

## Biochemical space language

The general goal of the language is to deal with the combinatorial explosion of numerous interactions by providing a concise and understandable notation. In order to capture fundamentally different features of biological objects, we define several types of *agents*. We show how we understand and use *rules* to describe the transformations of agents. Finally, we provide intuition behind the semantics of the rules. The full and formal definition is available in [43].

### Agents

In existing ontologies, objects residing in several different states (oxidised, reduced, etc.) are usually treated as separate objects, thus causing the total number of objects to be enormous. To reduce this complexity, the concept of *states* is introduced in BCS.

**Atomic agent** describes the most basic type of biological objects. Atomic agents are usually used to express small biological objects which can change their state. It holds information about the agent name and associated state.

For example, a carbonate in a 2- state, written CO_3{2-}, or a serine amino acid residue in a methylated state, Ser{met}.

**Structure agent** represents a biochemical object that is composed of several known atomic agents while we know that a composition is abstract and not necessarily complete. To incorporate this kind of abstraction into our language, a structure agent is defined to be labelled with a unique name. The key construct of a structure agent is *partial composition* defined as a set of atomic agents which are considered to be relevant parts of the structure agent. We allow this set to be empty with the meaning of a biological structure for which we do not know its composition.

For example, in a protein casein Cas two out of a few hundred amino acids (e.g. serine and tyrosine) are able to undergo some post-translational modifications, such as phosphorylation, methylation etc. It is suitable to model only these two acids instead of the entire primary structure of the protein. By Cas(Ser{p}, Tyr{u}) we express the fact that phosphorylated serine and unphosphorylated tyrosine are subparts of the protein Cas.

**Complex agent** represents a non-trivial composite biochemical object that is inductively constructed from already known biological objects. In rule-based languages, this is usually defined by introducing bonds between individual biochemical objects. In BCSL we abstract from the detailed specification of bonds and we rather assume a complex as a coexistence of certain objects in a particular group.

The key element of a complex agent is *sequence* describing inductively constructed coexistence expressions from existing agents. In contrast to partial composition in structure agent, we allow replication at the level of sequence (an agent of a certain name can appear more than once in a sequence). An example of a complex agent is a dimer of casein where all its amino acid residues are phosphorylated, written Cas(Ser{p}, Tyr{p}).Cas(Ser{p}, Tyr{p}).

The BCS is divided into **compartments**. Typical representatives on the level of molecular modelling are cell, cytosol, nucleus etc. Each complex agent belongs to one or several compartments. An atomic or a structure agent cannot belong directly to a compartment, but it is always part of a complex agent (the case when only one agent is in its sequence can occur). This guarantees each atomic and structure agent has indirectly given space location—the compartment. The previously given example has to be extended to the form Cas(Ser{p}, Tyr{p}).Cas(Ser{p}, Tyr{p})∷loc where loc can be any defined compartment.

Agents are associated with *signatures*, which describe allowed attributes for individual agents. There are three types of signatures. **Atomic signature** defines for an atomic agent allowed set of possible states. For example, for an atomic agent serine *Ser* it allows states methylated *met*, phosphorylated p, and neutral n. **Structure signature** defines for a structure agent allowed set of atomic agents. For example, for the casein, we define set of allowed atomic agents tyrosine Tyr and serine Ser. **Complex signature** allows replacement of a complex expression by an alias, which increases readability.

### Rules

**Fig 3:**
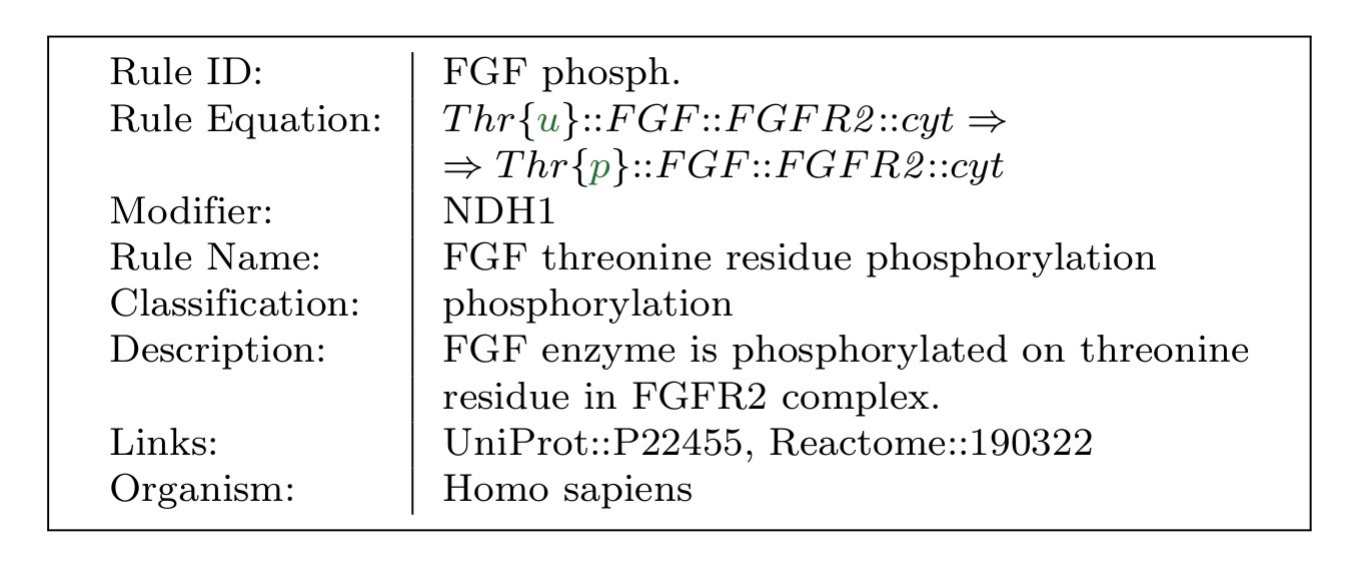
A rule employing agent state change. A threonine (Thr) amino acid residue of a FGF protein inside a FGFR2 complex can change its state from unphosphorylated u to phosphorylated p.

**Rule** is specified by *rule equation* enriched with additional annotation information (for an example, see Fig 3). When defining a rule equation, identifiers of *substrates* and *products* are used to make the notation of the rules compact. Every agent appearing in a rule equation has to be followed by the *localisation* operator associating it with a particular compartment. This is for example important for rules that act on both sides of a membrane. That way, a rule is always precisely localised in or between the compartments. A natural *stoichiometric* coefficient can be placed before any agent in a rule equation. Irreversible and reversible rules are distinguished by the operators ⇒ and ⇔ respectively. The + symbol is used as a separator between individual substrates and individual products. A rule can also have an assigned *classification*. Rule classification assigns a list of higher level biophysical processes in which the rule is involved.

In some cases, emphasis on detailed description leads to very complex BCS models. An abstraction of some processes is needed to keep BCS models as simple as possible. To this end, rules expressing enzymatic reactions are considered in a simplified form. In fact, there should be at least two different rules describing an enzymatic reaction (one for a substrate binding and another for a catalytic step). Instead, since an enzyme is not affected during the reaction, it is affiliated to the rule as a *modifier*. However, it is difficult to define the precise meaning of a modifier in this case. We rather treat the modifier attribute informally as an agent *which has to be present* for the rule to be enabled. The exact reaction mechanism of an enzyme is not always clear and therefore it is more rigorous to explicitly specify its function by rules.

The rule does not have precisely specify *which* agents undergo the transformation, it is possible to only specify restrictions on the agents. This is a key feature providing conciseness of the rule-based approach. An example describing the transformation of casein from the methylated serine residue to the neutral one can be expressed by the following rule:

Cas(Ser{met})∷cell⇒Cas(Ser{n})∷cell

It is important to notice that above we defined casein with two subparts Ser and Tyr, but tyrosine Tyr is not mentioned in the rule. It basically means its context has no influence on the process and therefore this action can happen regardless of the state of tyrosine.

The level of abstraction plays an important role in development of BCS. We will demonstrate this on a simple enzymatic scheme (Eq 1) and two fundamentally different approaches to model it.

$$\begin{array}{c} \begin{array}{c} E+S↔ES\\ ES\to E+P \end{array} \end{array}$$ (1)

This way we can model the enzymatic reactions associated with some rate laws quite precisely on the quantitative level [44]. However, when it comes to qualitative description, this form is not detailed enough. We can see some sort of equality between *E* + *S* and *E* + *P* which naturally leads to question what is the difference between *S* and *P*.

In order to capture such information, we could model these two agents as atomic agents with a shared name, differing only in the state (Eq 2). Please note we omit localisation information from the following rules only for simplicity, in all other cases it is mandatory.

$$\begin{array}{l} \text{E}+\text{R}\Leftrightarrow \text{E}.\text{R}\\ \text{E}.\text{R}\{\text{i}\}\Rightarrow \text{E}.\text{R}\{\text{a}\} \end{array}$$ (2)

In this case, we model both substrate S and product P as a reactant R, which can change its state from an inactive i to an active a if it is in the complex with enzyme E. The formation/disassembly of the complex can happen regardless of the particular state of the reactant. The reactant and its states can be annotated and the mechanism itself is more clear.

Another approach uses the fact that reactant is always involved in all rules, therefore plays a key role in modelling of this process. We can see the whole system as a structure agent R changing its features. In other words, we model the activity of reactant and the availability of enzyme as atomic agents residing in partial composition of the reactant (Eq 3).

$$\begin{array}{l} \text{R}(\text{active}\;\{\text{off}\},\;\text{enzyme}\;\{\text{avail}\})\;\Rightarrow \\ \Rightarrow \;\text{R}(\text{active}\;\{\text{on}\},\;\text{enzyme}\;\{\text{avail}\})\\ \text{R}(\text{enzyme}\;\{\text{avail}\})\;\Leftrightarrow \;\text{R}(\text{enzyme}\;\{\text{unavail}\}) \end{array}$$ (3)

The reactant R has two atomic agents (or *attributes*) which can change their states. Particularly, enzyme can change from available avail to unavailable unavail modelling presence/absence of the enzyme. Agent active might be either on or off, meaning the reactant is activated or deactivated, respectively. This way we could for example extend the reactant by some other attributes which are not influenced by these processes and model them independently.

We provide one more example dedicated to complex agents. As mentioned above, complex agents capture full enumeration of interacting subparts, which can be atomic or structure agents encapsulated in a compartment. However, it results in one obstacle for the compact syntax of the language—agents in its composition can be rather numerous. On the other hand, the process we want to express by a rule usually involves only a small fraction of the composition. For example, imagine that any protein Cas in the complex of eight same proteins (homo-octamer) can undergo phosphorylation on a serine Ser residue. Normally, we would have to enumerate the whole composition (Eq 4), where only one protein changed its state.

$$\begin{array}{l} \text{Cas}(\text{Ser}\;\{\text{u}\}).\text{Cas}.\text{Cas}.\text{Cas}.\text{Cas}.\text{Cas}.\text{Cas}.\text{Cas}∷\text{cyt}\Rightarrow \\ \Rightarrow \text{Cas}(\text{Ser}\;\{\text{p}\}).\text{Cas}.\text{Cas}.\text{Cas}.\text{Cas}.\text{Cas}.\text{Cas}.\text{Cas}∷\text{cyt} \end{array}$$ (4)

This form of description is neither concise nor easily readable. We can improve this notation by using information encoded in complex signatures—these associate complex agent name with its sequence. However, we cannot use it directly, we need to introduce nesting operator ‘∷’ between the agents (for simplicity it is identical with localisation operator). The main idea is to allow zooming into individual parts of complex and structure expressions.

Ser{u}∷Cas∷Cas8∷cyt⇒Ser{p}∷Cas∷Cas8∷cyt (5)

Eq 5 has the same meaning as the previous equation with the assumption we have defined complex signature with name Cas8 (which can be arbitrary). It allows the change of state of one of the Cas proteins inside the complex.

### Semantics

After a brief description of the language and its practical impact, we need to understand the exact semantic meaning of the rules. The precise mathematical definition is provided in [43]. Compared to the first version of the language, the definition was reformulated in terms of set theory contrary to the original process algebra approach. This allows a more natural formulation of modelled in objects in biological context. In this section we describe the semantics on an informal level using a schematic approach, which is both intuitive and demonstrative.

By square and circle we refer to different types of atomic/structure agents and by colour to its state/feature.

**Fig 4:**
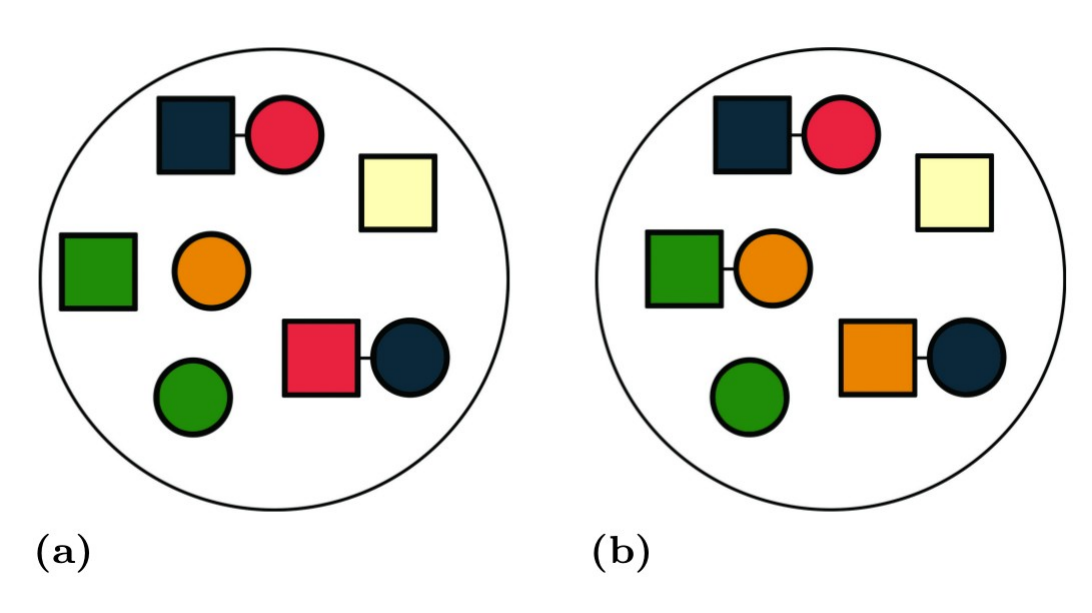
Examples of solutions. (A) An example of graphical representation of a solution. (B) Updated solution after the rules from Fig 5 were applied. The first rule Fig 5A was applied on the green square and the orange circle and produced complex square-circle complex. Note there are more options how the agents could be matched by the rule—each combination of free square and circle. The second rule Fig 5B was applied on the complex of red square and blue circle where the colour of square was changed from red to orange. The third rule Fig 5C could not be applied because there is no such complex with a green circle.

We represent a BCSL model as a set of rules and an *initial solution* of interacting agents. We understand the solution as a mixture of individual agents which are randomly distributed (an example is given in Fig 4A). Therefore, we cannot assign them any order and we represent them as multisets. From a biological perspective, this representation of the solution is as close as is possible to reality (neglecting spacial information) while preserving conciseness.

**Fig 5:**
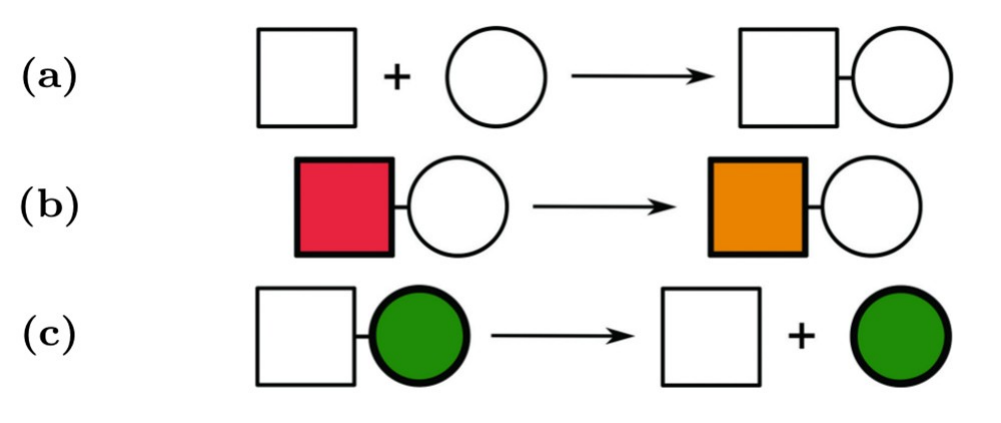
Examples of rules. Rule (A): a square and a circle can form a complex regardless their colours. Rule (B): a square is allowed to change its colour from red to orange only if it is in a complex with a circle regardless its colour. Rule (B): the rule can disassemble the complex only if the circle is green.

**Fig 6:**
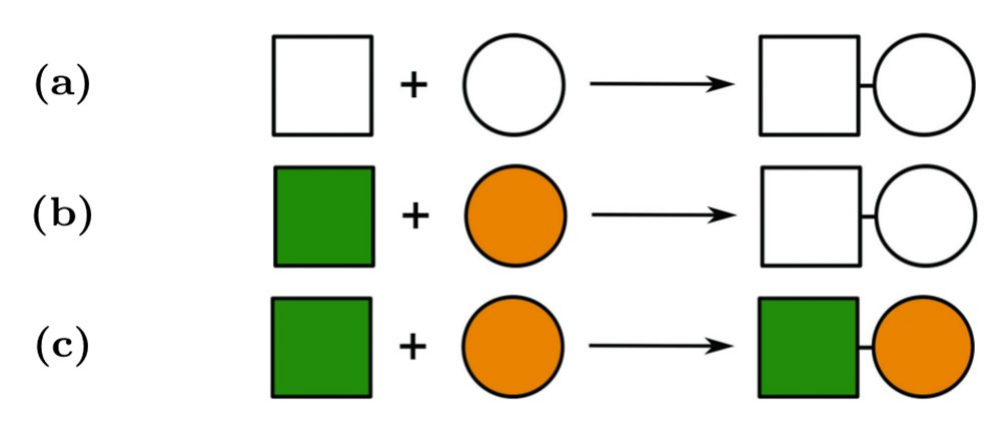
Example of a match-replace action. As a solution, we use the solution A from Fig 4. (A) Rule can match a square and a circle regardless their colours and form a complex. (B) We randomly choose the green square and the orange circle from the solution. The rule matched the chosen objects and they were assigned to the left-hand side of the rule. (C) The rule was applied and objects we replaced by a new complex of green square and orange circle. We obtained the reaction describing the particular action which has just happened.

The rules are patterns which describe the behaviour of groups of agents. A rule has the form of an abstract chemical reaction, where substrates and products take place. The difference is that a reaction only operates on particular agents, while in the rule groups of agents are interacting. Therefore, the reaction (e.g. Fig 6C) can be seen as a special case of the rule, where the type represents exactly one object.

The rule is mapped on a solution (process called *matching*) and then it can be applied (process called *replacement*) and a new solution is produced. The matching is not always successful (Fig 4, application of rule C). The matching can be seen as assigning particular objects from the solution to types on the left-hand side of the rule.

The replacement represents the change of the matched objects to new objects according to the right-hand side of the rule (i.e. the particular objects are assigned to the right-hand side). As a by-product, we obtain an instance of the rule—a reaction (Fig 6).

Definition of the semantics naturally gives us the possibility to generate transition systems for a model. It allows numerous options for dynamic analysis, particularly model checking [45], which allows us to reason about properties of the models and their verification.

### Analysis

The BCSL offers interesting features that allow static analysis of given models. In this section, we provide intuition behind these types of analysis and explain their practical impact in the case study. Their formal definition is available in [43].

Analyses presented in this section are based on *compatibility* relation defined on agents. Intuitively, this relation formulates the level of difference in specificity between the agents, e.g. Cas(Ser{p}, Tyr{u}) is more specified than Cas(Tyr{u}), because in the latter the state of Ser is not given.

Atomic agent A is compatible with B if their states are either equal or state of A is less specified, i.e. not given.

In the case of structure agents (example above), agent A is compatible with B if its partial composition is less specified. More precisely, for each atomic agent in A there exists an atomic agent in B such that they are compatible.

Compatibility of complex agents is very similar to the case of structure agents, but we have to consider any possible permutation of the sequences since the order does not matter.

**Fig 7:**
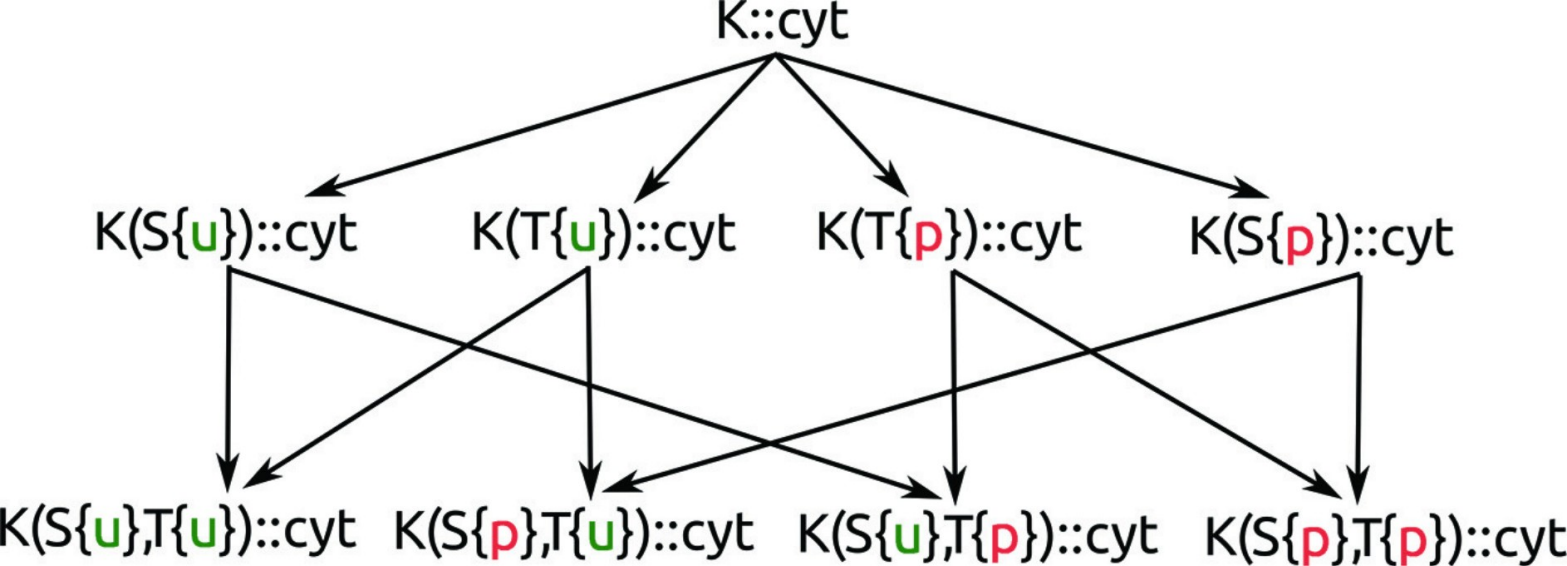
An example of agents partially ordered by compatibility relation. It is formed by a complex in cyt compartment, which has only one structure agent K in its sequence. The structure agent K has atomic agents T and S allowed in its partial composition. They might occur in two states—u and p. All relevant agents are interconnected by compatibility relation.

The defined compatibility naturally forms partial order on the agents, which enables comparison of the agents (for an example, see Fig 7).

#### Rule redundancy elimination

There might be redundant rules defined in a model. Redundant means that semantically equal or more general rule already exists in the model. This might be done for example by the inattention of modeller in combination with high level of abstraction the language uses. These rules do not cause any semantic difference, but can possibly slow down dynamic and/or static analysis, eventually undesirably affecting the results of analysis.

These redundant rules can be identified and consequently eliminated from the model using compatibility relation and the fact that if every two agents from two rules on the same position are compatible, then we can declare the whole rule as compatible with the other one and it is semantically less general, i.e. does not add any information.

We will demonstrate this fact on the following example, where we derive all possible reactions from the given rules (associated with signatures) and show that there is a relation between the sets.

Let us have two rules (for simplicity, we omit compartment, which would be the same for all agents in all rules):

K(S{u}).K()⇒K(S{p}).K() (6)

K(S{u}),T{i}.K()⇒K(S{p},T{i}).K() (7)

The rules describe change of state for a dimer of K agents. This happens on an atomic agent S in both cases. However, in case of the second rule, the context of application is additionally restricted to presence of atomic agent T in state i.

Considering structure signature K → {S, T} and atomic signatures S → {u, p} and T → {a, i}, the rule (6) produces the following set of eight reactions:

$$\begin{array}{l} \text{K}(\text{S}\;\{\text{u}\},\text{T}\;\{\text{a}\}).\text{K}(\text{S}\;\{\text{u}\},\text{T}\;\{\text{a}\})\;\Rightarrow \;\text{K}(\text{S}\;\{\text{p}\},\text{T}\;\{\text{a}\}).\text{K}(\text{S}\;\{\text{u}\},\text{T}\;\{\text{a}\})\\ \text{K}(\text{S}\;\{\text{u}\},\text{T}\;\{\text{a}\}).\text{K}(\text{S}\;\{\text{u}\},\text{T}\;\{\text{i}\})\;\Rightarrow \;\text{K}(\text{S}\;\{\text{p}\},\text{T}\;\{\text{a}\}).\text{K}(\text{S}\;\{\text{u}\},\text{T}\;\{\text{i}\})\\ \text{K}(\text{S}\;\{\text{u}\},\text{T}\;\{\text{a}\}).\text{K}(\text{S}\;\{\text{p}\},\text{T}\;\{\text{a}\})\;\Rightarrow \;\text{K}(\text{S}\;\{\text{p}\},\text{T}\;\{\text{a}\}).\text{K}(\text{S}\;\{\text{p}\},\text{T}\;\{\text{a}\})\\ \text{K}(\text{S}\;\{\text{u}\},\text{T}\;\{\text{a}\}).\text{K}(\text{S}\;\{\text{p}\},\text{T}\;\{\text{i}\})\;\Rightarrow \;\text{K}(\text{S}\;\{\text{p}\},\text{T}\;\{\text{a}\}).\text{K}(\text{S}\;\{\text{p}\},\text{T}\;\{\text{i}\})\\ \text{K}(\text{S}\;\{\text{u}\},\text{T}\;\{\text{i}\}).\text{K}(\text{S}\;\{\text{u}\},\text{T}\;\{\text{a}\})\;\Rightarrow \;\text{K}(\text{S}\;\{\text{p}\},\text{T}\;\{\text{i}\}).\text{K}(\text{S}\;\{\text{u}\},\text{T}\;\{\text{a}\})\\ \text{K}(\text{S}\;\{\text{u}\},\text{T}\;\{\text{i}\}).\text{K}(\text{S}\;\{\text{u}\},\text{T}\;\{\text{i}\})\;\Rightarrow \;\text{K}(\text{S}\;\{\text{p}\},\text{T}\;\{\text{i}\}).\text{K}(\text{S}\;\{\text{u}\},\text{T}\;\{\text{i}\})\\ \text{K}(\text{S}\;\{\text{u}\},\text{T}\;\{\text{i}\}).\text{K}(\text{S}\;\{\text{p}\},\text{T}\;\{\text{a}\})\;\Rightarrow \;\text{K}(\text{S}\;\{\text{p}\},\text{T}\;\{\text{i}\}).\text{K}(\text{S}\;\{\text{p}\},\text{T}\;\{\text{a}\})\\ \text{K}(\text{S}\;\{\text{u}\},\text{T}\;\{\text{i}\}).\text{K}(\text{S}\;\{\text{p}\},\text{T}\;\{\text{i}\})\;\Rightarrow \;\text{K}(\text{S}\;\{\text{p}\},\text{T}\;\{\text{i}\}).\text{K}(\text{S}\;\{\text{p}\},\text{T}\;\{\text{i}\}) \end{array}$$

while the rule (7) produces the set of four reactions which is a subset of the previous one:

$$\begin{array}{l} \text{K}(\text{S}\;\{\text{u}\},\text{T}\;\{\text{i}\}).\text{K}(\text{S}\;\{\text{u}\},\text{T}\;\{\text{a}\})\;\Rightarrow \;\text{K}(\text{S}\;\{\text{p}\},\text{T}\;\{\text{i}\}).\text{K}(\text{S}\;\{\text{u}\},\text{T}\;\{\text{a}\})\\ \text{K}(\text{S}\;\{\text{u}\},\text{T}\;\{\text{i}\}).\text{K}(\text{S}\;\{\text{u}\},\text{T}\;\{\text{i}\})\;\Rightarrow \;\text{K}(\text{S}\;\{\text{p}\},\text{T}\;\{\text{i}\}).\text{K}(\text{S}\;\{\text{u}\},\text{T}\;\{\text{i}\})\\ \text{K}(\text{S}\;\{\text{u}\},\text{T}\;\{\text{i}\}).\text{K}(\text{S}\;\{\text{p}\},\text{T}\;\{\text{a}\})\;\Rightarrow \;\text{K}(\text{S}\;\{\text{p}\},\text{T}\;\{\text{i}\}).\text{K}(\text{S}\;\{\text{p}\},\text{T}\;\{\text{a}\})\\ \text{K}(\text{S}\;\{\text{u}\},\text{T}\;\{\text{i}\}).\text{K}(\text{S}\;\{\text{p}\},\text{T}\;\{\text{i}\})\;\Rightarrow \;\text{K}(\text{S}\;\{\text{p}\},\text{T}\;\{\text{i}\}).\text{K}(\text{S}\;\{\text{p}\},\text{T}\;\{\text{i}\}) \end{array}$$

This way it is shown that the rule (7) is more specified than the rule (6) and therefore does not add any additional semantic information for the model, i.e. it is redundant. In other words, by removal of the rule the qualitative behaviour of the model it is included in does not change.

#### Context-based reduction

There might be cases when eliminating context of the given BCSL model preserves some properties while making the analysis of the model simpler. This is particularly case of dynamic analysis, where minor change in the model specification can dramatically affect the behaviour.

This context elimination is enabled by changing all specified states of agents to unspecified. Formally it is done using compatibility relation by choosing the least specified agent as illustrated in Fig 7. A reduced model is generated from the original model and allows us to reason about some properties which are preserved. For example, some reachability properties can be answered in the reduced model. An example can be found in Case study.

#### Static non-reachability analysis

The compatibility relation can be used for static non-reachability analysis before enumerating the entire transition system of the model. This is done by specifying the following property: an agent is non-reachable if for all agents on the right-hand side of any rule holds that they are not compatible with the given agent (except the trivial case when the agent is already present in the initial state).

The source for arguments of this statement is based on the fact that in order to reach an agent, there has to be a rule which creates the same or at least a compatible agent. It ensures that the particular states of an agent (or its subparts) are changed at some point. However, it does not ensure the reachability of the agent (because the question whether the combination of multiple rules is applicable is more complicated), but can eliminate the possibility of opposite property—the non-reachability. For example, we cannot expect a protein Cas with phosphorylated serine to be reachable from the unphosphorylated state if there is not a rule of form

Cas(Ser{u})⇒Cas(Ser{p})

in the model. An example of the analysis application is given in Case study.

## Case study

BCS is currently implemented in e-cyanobacterium.org [35], a web-based platform for the modelling and analysis of biological processes occurring in cyanobacteria. The platform provides several features that contribute to the production and presentation of models targeting cyanobacteria. The principal effort is to interlink biological knowledge with the benefits of computational systems biology tools. As an instance of CMP, it covers all the features of the platform.

Various environmental and cellular processes are present in BCS, such as photosynthesis, carbon concentrating mechanism, circadian clock, and metabolism. All of them are covered in corresponding models, each of them properly mapped on the BCS. Entire biochemical space of cyanobacteria is formed from over a thousand agents and several hundred rules. From the field of cyanobacteria modelling, we provide several cases which demonstrate the application of selected BCSL features to specification of particular biological processes, their annotation, and analysis. In addition, we demonstrate the usage of our framework outside of the cyanobacteria domain by describing and analysing a signalling pathway appearing in mammalian cells.

### Model specification

The circadian clock of cyanobacteria [46, 47] is formed by three proteins KaiA, KaiB, and KaiC (with additional presence of ATP). Such oscillator composed of three Kai proteins and ATP can be reconstituted *in vitro*, making it the simplest post-translational circadian oscillator currently known [48]. The key role is played by KaiC, which naturally forms a homohexamer. Compared to the other two proteins, it is physically the largest one. KaiC contains two phosphorylation sites important for the modelling—threonine 432 and serine 431 [49] (UniProt∷Q79PF4). These two amino acids can be modified by phosphorylation. However, the sites are accessible only when the protein forms a hexamer. The KaiA, forming a dimer, binds to the KaiC hexamer and promotes KaiC phosphorylation, whereas the effect of KaiB binding to the KaiC hexamer is opposite as it stimulates KaiC dephosphorylation [50–52]. There are several complexes which can be formed from mentioned proteins according to the current situation with the KaiC hexamer.

It is important to note the KaiA and KaiB proteins only *enhance* (resp. *suppress*) the phosphorylation process, but do not enable (resp. disable) it completely [53] (except the complex with six KaiB proteins when the phosphorylation domains are basically inaccessible). When it comes to the quantitative description of the system, it is necessary to depict all possible actions. Particularly, we need to express the fact that the phosphorylation process happens regardless of the particular complex KaiC protein is currently in. This is defined by a rule

S{u}∷KaiC∷?X∷cyt⇔S{p}∷KaiC∷?X∷cyt

$$?\text{X}=\{\begin{array}{c} \text{KaiC}6,\;\text{KaiA}2\text{C}6,\;\text{KaiA}4\text{C}6,\\ \text{KaiA}4\text{B}6\text{C}6,\;\text{KaiA}6\text{B}6\text{C}6 \end{array}\}$$

where phosphorylation of serine residue is possible for all complexes with KaiC included. This is denoted by a variable ?X is used in place of a complex.

An interesting aspect of the cyanobacterial circadian clock mechanism is the formation of KaiC hexamers. The condition that the KaiC protein can be (de)phosphorylated mostly inside a hexamer leads to one important fact—it does not mean the hexamer must be assembled from (resp. dissociated to) unphosphorylated KaiC proteins. The hexamer can be dissociated at any point in time, for example when three KaiC proteins are phosphorylated on the serine residue, other two are phosphorylated on both residues, and the last one is not phosphorylated at all. The number of possible combinations is quite large.

Rule-based languages, including BCSL, are very suitable for such cases. What we actually need to express is the situation when a complex is formed (or dissolved), regardless of the context (particular states) of the interacting agents. In a rule

6KaiC∷cyt⇔KaiC6∷cyt

the formation of KaiC hexamer from six KaiC proteins and its dissociation are expressed. The rule requires to have a defined complex signature KaiC6. The complex can be formed (resp. dissolved) regardless of the context of individual KaiC proteins.

Another important process inside cyanobacteria is photosynthesis, occurring on thylakoids membranes. In general, photosynthesis in cyanobacteria uses water as an electron donor and produces oxygen as a product. Electron transport is performed through multiple protein complexes, which together form the thylakoid membrane itself. Such complexes are for example photosystem I and II. However, direct modelling of the complexes is not very efficient. The typical rules in photosynthesis are just electron transfers between the complexes or reduction/oxidation of individual parts. Therefore, such action usually happens regardless of the context of the rest of the complexes in a photosystem. The best approach with respect to BCSL language is to use structure agent for defining the protein complexes and its individual parts model as partial composition. This, for example, is expressed in a rule

ps2(oec{2+},yz{+})∷tlm⇔ps2(oec{3+},yz{n})∷tlm

describing the oxidation of S2-state of the oxygen-evolving complex oec{2+} by Yz{+} in photosystem II. There are many more subparts of photosystem II. (e.g. chlorophyl, pheophytin), but their states are not important in this context.

The last example targets carbon dioxide concentrating mechanism (CCM). The CCM of cyanobacteria consists of structural enzymes and proteins that enable the increase of the local concentration of CO_2 around the carbon-fixing enzyme RuBisCO (ribulose-1,5-bisphosphate carboxylase/oxygenase) up to three orders of magnitude. Cyanobacterial growth in a native aqueous environment with low concentrations of CO_2 is enabled by the mechanism. An important part of this mechanism is active transport of carbon in the form of hydrogen carbonate inside the cell enabled by sodium. It is described by a rule

HCO_3{−}∷ext+Na{+}∷ext⇒HCO_3{−}∷cyt+Na{+}∷cyt

expressing the transport of hydrogen carbonate from extracellular space inside the cytosol of the cell. This transport is enabled by presence of sodium cations.

### Annotation

The concrete BCS constructed for a given biological problem plays an important role in the annotation of mathematical models. The particular goal is to assign the models their biological meaning. The annotation is performed by means of a *mapping* of functional parts of the model to relevant parts of BCS. We will show this on an example related to the circadian clock from the previous section.

An example of a circadian clock model of cyanobacteria is [53], which describes the two-loop transcriptional feedback mechanism. The crucial step in the implementation of the model on e-cyanobacterium.org has been its annotation. This process has raised several questions on the details about the structure of the model.

Particularly, the participation of KaiB in the interactions has been omitted, which caused a severe confusion in the understanding of the mechanism behind the process of circadian protein complexes formations. This issue has been resolved by mapping the model equations to the BCS complex formation rules resulting in precise mechanistic description of the involved interactions.

The model uses possible complex forms organised as so-called *pools*, which couples a class of agents of particular forms. The pools play an important role in phosphorylation processes and can be naturally expressed using the rule-based approach. These processes could be then directly mapped on some variants of phosphorylation BCS rules from the example in Model specification section.

Another example of a circadian clock model is [54], which uses a different level of abstraction. Instead of pools, the model assumes *partially* (some of the active sites) and *completely* (all of the active sites) phosphorylated hexamer forms.

Therefore any phosphorylation step can be mechanistically represented by two possible actions—either KaiC is phosphorylated on the serine or on the threonine residue. Every such model equation has been mapped to both BCS phosphorylation rules (for details see Model repository).

Finally, we have compared both models at the level of BCS. In this step, some critical conflicts and overlaps have been detected. For example, we have found a significant difference in the regulation mechanism of KaiB tetramers. The abstraction employed by [53] model omits the KaiB completely and implicitly assumes the proteins as constant, therefore tetramers are not formed. On the other hand, in [54] model, these tetramers are formed explicitly since they play an important role in the regulation of KaiC complexes dephosphorylation.

### Analysis

We demonstrate the practical purposes of a few types of static analysis which are enabled by interesting features of the language. These can be used to analyse BCS during its construction and help to reveal non-trivial issues. Their formal definition is available in [43].

For this purpose, we use *fibroblast growth factor* (FGF) signalling pathway employed in mammalian cells. Our example is based on [55] model. On this example, we also show that processes on a different level of abstraction and different domain can be effectively described using BCSL.

The entire signalling pathway consists of 20 agents interacting in 57 rules (for details, see [43]. Most of the proteins can undergo phosphorylation (represented by a state change from u to p on some amino acid residues). We consider initial conditions such that there are all required agents in one or two repetitions (some complexes require multiplicity). In such a case, the number of reachable states can grow up to 2^72^, which is too high to be effectively enumerated.

For example, the goal is to check whether given complex agent FRS(Thr{u},Tyr{u}).FGF(Thr{u}).R.FGF(Thr {u}).R∷cyt is a reachable agent for the given model. The agent is formed from FGF proteins which are unphosphorylated (u) on threonine residues (Thr). We check if the agent is non-reachable using the *static reachability analysis*, which can answer some reachability properties without the execution of models behaviour. The conclusion is that there is no compatible agent on any right-hand side of the rules. It follows that the given complex agent is non-reachable.

The *context-based reduction* is demonstrated on the same pathway as in the previous case. It produces an over-approximation of modelled system. The resulting model has 16 bidirectional rules (for details, see [43]). The size of the transition system has significantly decreased—it has approximately six hundreds of states and two thousands of edges.

For the demonstration, we check the reachability of a complex agent Raf(Thr{p}).ERK(Tyr{p},Thr{p})∷cyt. Using the reduced model, we can first check whether its corresponding least specified agent Raf.ERK::cyt is non-reachable. Since the transition system of the model is relatively small, it can be quite easily done by exploring the transition system. The answer is non-reachable which means the original agent is non-reachable too.

## Comparison

In this section, we compare BCSL to the most dominant rule-based languages regarding some key features. In doing so, we use the circadian clock of cyanobacteria introduced in Model specification section of the case study as a running example. We focus on the mechanism of auto-(de)-phosphorylation of the KaiC protein inside a homohexamer (UniProt∷Q79PF4). We have selected BioCHAM [23], Kappa [28], and BNGL [10] as three rule-based representatives for this comparison, as they cover the most important features found in all rule-based languages.

There are several features which are present in all four languages, such as the definition of agents with an internal structure, which is equipped by well-defined states. Such agents can interact in rules with each other (e.g. formation of complexes) or change their internal structure (e.g. a particular state). However, the particular implementation of these features can differ in individual formalisms. Moreover, there are some features, such as compartmentalisation or binding, which are not used in all the compared languages.

### Agents structure

Defining agents that have an internal structure has several advantages. It might be useful in biological modelling as it allows expressing the structure of biochemical objects more precisely. It also becomes beneficial when writing rules since the structure allows skipping the irrelevant context.

In BCSL, we have defined three types of agents—atomic, structure, and complex. Atomic agents can be used to represent *features* of structure agents, but can also be used independently. For details, see Agents section. In Kappa and BNGL, there exists only one type of agent. This agent can have defined *sites* with associated state and bond (see Complex representation).

For example, suppose we want to express KaiC protein phosphorylated on serine residue S and unphosphorylated on threonine residue T. This can be expressed in BCSL as KaiC(S{p},T{u}). We can do something similar in BNGL and Kappa—KaiC(S∼p, T∼u)—which, in this case, looks the same in both languages. S and T are sites in particular states. Syntactically all three languages are very similar in this aspect. However, it is worth noting that in BCSL, atomic agents used inside structure agent are understood as individual (atomic) entities which allows to use them in rules separately. Furthermore, they can be assigned individual annotation records.

Agents can also be *flat* with no explicitly defined structure. Flat agents do not bring on the advantages mentioned above, but can be considered as a simpler notation in some cases. Flat agents are used in BioCham. The agents have no internal structure; they have assigned a set of states only. Therefore, any structural details captured in formalisms described above have to be encoded in states. The particular encoding is up to the modeller since there are multiple options on how to express the four possible conformations of the KaiC protein. For example, we can define a unique state for every option or use the fact that we can assign a set of states (possibly empty) to the agent. Then, we can write KaiC∼{S_p} to express the particular conformation from our example.

### Complex representation

Complex formation is a way how individual agents can interact and exchange information. On this level, we recognise two types of complexes—with explicit and implicit binding—and two approaches how complexes are matched by the rules—full or partial match.

Implicit binding does not allow expressing how exactly the agents are connected. This is the case of BCSL, where a complex of six KaiC proteins is given by the expression KaiC().KaiC().KaiC().KaiC().KaiC().KaiC()∷cyt where the particular meaning of the complex is *coexistence*, not implying what kind of physical interaction does it represent (the role of compartment cyt is discussed in Compartmentalisation). In BioCham, the meaning is similar, but the notation for complex is different: KaiC-KaiC-KaiC-KaiC-KaiC-KaiC.

Explicit binding, on the other hand, allows defining how, in particular, the agents are connected and represent a physical connection. It offers interesting modelling possibilities such as the definition of internal changes on a structural level (e.g. protein folding). Explicit binding is enabled in BNGL and Kappa by connecting pairs of sites by a bond identifier. For example, KaiC(m!1).KaiB(n!1) represents two BNGL agents connected through their sites m and n by a bond identified by number one. The example is almost identical for Kappa except they do not use a special identifier for complex, the agents are just delimited by a comma. An important difference is that in BNGL it is allowed for one site to have multiple bonds (e.g. KaiC(m!1!2)), while this is not allowed in Kappa. Moreover, in BNGL, two sites in one agent can have the same name; this is not permitted in Kappa.

However, despite many advantages of the explicit approach, in the case of our running example, it complicates the way some properties can be modelled. The information that the six KaiC proteins are connected is not enough to express the particular bonds. For example, it could be written like a circular complex in BNGL KaiC(m!1!2).KaiC(m!2!3).KaiC(m!3!4).KaiC(m!4!5).KaiC(m!5!6).KaiC(m!6!1). In Kappa, this is even more complicated since we need to introduce at least two sites to each agent to form bonds with additional two proteins. Other options would be for example a linear conformation or a complete graph—particular bonding have to be validated and such information is often difficult to obtain in structural chemistry. Simply put, in this case, we have to deal with details which are not important for our modelling goals. On the other hand, there are modelling cases when the implicit binding is not enough, such as structural changes inside molecules.

Complexes modified by rules can be matched as whole objects or it is possible to allow local changes. In the case of BCSL and BioCham, both languages employ *full* match approach. A rule changing protein KaiC in BCSL

KaiC(S{u})∷cyt⇒KaiC(S{p})∷cyt

does match only single KaiC proteins, it cannot match any of KaiCs in the hexamer complex. It is necessary to enumerate the whole complex we want to be modified by the rule. With the addition of using nesting, variables, and complex aliases, we can write compact rules matching multiple complexes (for an example, see the first rule in Model specification section).

In Kappa and BNGL, it is possible to write rules similarly, as explained above. Moreover, rules with *partial* match focusing only on local context can also be specified. We show this approach on BNGL since Kappa has an analogous mechanism for this feature. The three rules

$$\begin{array}{l} \text{KaiC}\;(\text{m}\;!\;+,\text{S}\sim \text{u})\to \text{KaiC}\;(\text{m}\;!\;+,\text{S}\sim \text{p})\\ \text{KaiC}\;(\text{m},\text{S}\sim \text{u})\to \text{KaiC}\;(\text{m},\text{S}\sim \text{p})\\ \text{KaiC}\;(\text{S}\sim \text{u})\to \text{KaiC}\;(\text{S}\sim \text{p}) \end{array}$$

express a change of state of KaiC agent with a mandatory bond (denoted by + sign), no bond (site m is empty), and optional bond (site m is not specified), respectively. The first rule can be applied to any KaiC agent which is in a complex. It could be used as an alternative to the first BCSL rule from Model specification section. However, the phosphorylation is not allowed for complex KaiB6C6, so the rule can match more complexes than is intentional. The second rule has the same meaning as the BCSL rule above. Finally, the third rule is a combination of the other two cases since it has no restrictions on the particular binding of site m.

Moreover, the partial match type of rules can cause so-called *side effects*. For example, degradation of an agent with mandatory bond disconnects this bond from another agent which is not explicitly used in the rule—thus the side effect of the rule. While we think this feature increases the expressive power of formalisms it is supported by (it allows, for example, writing polymerisation rules), on the other hand, it offers an opportunity to unintentionally write rules which influence the network by undesired behaviour.

### Compartmentalisation

The last feature we consider is related to the modelling of spacial information about the agents. This is in particular useful in the context of systems biology as many biological objects travel through many locations inside the cell or participate in cell signalling. Moreover, such interactions occur on multiple organisational levels of biological systems [56].

Spatial description can be added to a reaction network model by defining compartments. Compartments represent sections of the modelled system separated by a physical (e.g. cell membrane) or functional (e.g. DNA interaction in prokaryotes) barrier. They can be further refined by defining compartment geometries and explicitly representing the species concentrations as a function of position [57]. Another approach is to track spatial position of every agent in the system and model their dynamics explicitly [58]. However, modelling the particular location directly (e.g. position in 3D space) is computationally difficult and such an approach is often over-complication (yet there are cases when it is necessary).

To the best of our knowledge, there is no support for compartments in BioCham. BNGL provides a compartmental extension [59, 60]. It allows to define a hierarchy of surface (e.g. membrane) and volume (e.g. cytoplasm) compartments and localise individual agents within them. Complexes can comprise agents in adjacent compartments, but they can span at most one surface and two adjacent volume compartments. The localisation of agents separately is allowing partial transport of complexes. It is also possible to explicitly specify to transport all connected agents.

Kappa also provides an extension [61], which supports spatial concepts in the form of voxel-based compartments, channels (connections between compartments), and translocation of species. Complex species are allowed to span multiple compartments. Compared to BNGL compartmental extension, spatial Kappa does not assume well-stirred contents of compartment and allows it to be distributed heterogeneously.

In BCSL, compartments are mandatory for all agents and allow expressing rules such as transport and help to separate the influence of rules in individual locations. Compared to BNGL, implementation of compartments is simpler; it is not possible to define relations among compartments. Moreover, partial localisations within complexes are not allowed since the compartments are directly associated with whole complex agents.

## Discussion

We have presented the second generation of BCSL, a novel high-level language for the hierarchical description of biological structures and mechanistic description of chemical reactions by means of compact rules. With respect to the previous prototype [33] the framework fully utilises the specific view on the biochemical structures and reactions by means of direct semantics.

The case studies have shown the presented version of BCSL fulfils our aims to use a formal specification aside of ODE models. We have demonstrated that the BCS mapping can significantly improve the understanding of modelled biological processes. Constructing the consistent BCS makes a key step in the process of integrating individual models as shown in [62].

The main advantages of the presented language are structured organisation of agents providing a natural description of biological objects; bondless formation of complexes with meaning of coexistence giving the modeller its custom interpretation, reducing the problem of combinatorial explosion; explicit complex manipulation avoiding misinterpretations of rules and their side effects; and support of compartments to model spatial information often playing an important role in modelling of biological systems.
